# Copy Number Alteration and Mutational Profile of High-Grade B-Cell Lymphoma with *MYC* and *BCL2* and/or *BCL6* Rearrangements, Diffuse Large B-Cell Lymphoma with *MYC*-Rearrangement, and Diffuse Large B-Cell Lymphoma with *MYC*-Cluster Amplification

**DOI:** 10.3390/cancers14235849

**Published:** 2022-11-27

**Authors:** Masashi Miyaoka, Yara Yukie Kikuti, Joaquim Carreras, Atsushi Ito, Haruka Ikoma, Sakura Tomita, Hiroshi Kawada, Giovanna Roncador, Silvia Bea, Elias Campo, Naoya Nakamura

**Affiliations:** 1 Department of Pathology, School of Medicine, Tokai University, 143 Shimokasuya, Isehara 259-1193, Kanagawa, Japan; 2 Department of Hematology/Oncology, School of Medicine, Tokai University, 143 Shimokasuya, Isehara 259-1193, Kanagawa, Japan; 3 Monoclonal Antibodies Unit, Spanish National Cancer Research Center (Centro Nacional de Investigaciones Oncologicas, CNIO), Melchor Fernandez Almagro 3, 28029 Madrid, Spain; 4 Hematopathology Section, Molecular Pathology Laboratory, Department of Pathology, Hospital Clinic Barcelona, Institut d’Investigacions Biomediques August Pi i Sunyer (IDIBAPS), Centro de Investigacion Biomedica en Red de Cancer (CIBERONC), University of Barcelona, C. de Villarroel, 170, 08036 Barcelona, Spain

**Keywords:** high-grade B-cell lymphoma with MYC and BCL2 and/or BCL6 rearrangements, double/triple-hit lymphoma, single-hit lymphoma, MYC-cluster amplification, copy number alterations, mutational profile

## Abstract

**Simple Summary:**

Diffuse large B-cell lymphoma (DLBCL) is one of the most frequent non-Hodgkin lymphomas. DLBCL with *MYC* alteration is classified as (1) high-grade B-cell lymphoma with *MYC* and *BCL2* and/or *BCL6* rearrangements (double/triple-hit lymphoma; DHL/THL), (2) DLBCL with *MYC* rearrangement (single-hit lymphoma; SHL), and (3) DLBCL with *MYC*-cluster amplification (MCAD). This research analyzed these three lymphoma subtypes using an integrative approach, including in situ hybridization (FISH), whole-genome copy number, and targeted next-generation sequencing (NGS). There are differences between them.

**Abstract:**

Diffuse large B-cell lymphoma (DLBCL) with *MYC* alteration is classified as high-grade B-cell lymphoma with *MYC* and *BCL2* and/or *BCL6* rearrangements (double/triple-hit lymphoma; DHL/THL), DLBCL with *MYC* rearrangement (single-hit lymphoma; SHL) and DLBCL with *MYC*-cluster amplification (MCAD). To elucidate the genetic features of DHL/THL, SHL, and MCAD, 23 lymphoma cases from Tokai University Hospital were analyzed. The series included 10 cases of DHL/THL, 10 cases of SHL and 3 cases of MCAD. The analysis used whole-genome copy number microarray analysis (OncoScan) and a custom-made next-generation sequencing (NGS) panel of 115 genes associated with aggressive B-cell lymphomas. The copy number alteration (CNA) profiles were similar between DHL/THL and SHL. MCAD had fewer CNAs than those of DHL/THL and SHL, except for +8q24. The NGS profile characterized DHL/THL with a higher “mutation burden” than SHL (17 vs. 10, *p* = 0.010), and the most relevant genes for DHL/THL were *BCL2* and *SOCS1*, and for SHL was *DTX1*. MCAD was characterized by mutations of *DDX3X*, *TCF3*, *HLA-A*, and *TP53*, whereas *MYC* was unmutated. In conclusion, DHL/THL, SHL, and MCAD have different profiles.

## 1. Introduction

When diffuse large B-cell lymphoma (DLBCL) has *MYC* rearrangement (*MYC*-R) and additional *BCL2*-R and/or *BCL6*-R, called double/triple-hit lymphoma (DHL/THL), the lymphoma generally has a highly adverse prognosis [1,2,3,4]. These findings introduced a new category of “High-grade B-cell lymphoma with *MYC* and *BCL2* and/or *BCL6* rearrangements” to the World Health Organization 2017 Classification of Tumors of Hematopoietic and Lymphoid Tissues [5]. However, Ennishi et al. and our group independently reported that the overall survival (OS) curve of DLBCL with a double-hit gene expression signature and DHL/THL showed similarities to the activated B-cell-like (ABC) DLBCL [6,7]. These findings indicated that some patients with DHL/THL achieved a favorable clinical outcome with the usual R-CHOP therapy.

Initially, DLBCL with *MYC*-R but without *BCL2*-R and *BCL6*-R (single-hit lymphoma; SHL) was reported to be associated with an adverse prognosis. Rosenwald et al., however, recently found that the negative prognostic impact of *MYC*-R was observed in patients with DLBCL within only the first 24-month follow-up [8].

Currently, there are conflicting reports on the genetic features of DHL/THL [9,10,11,12,13,14,15]. Some studies have demonstrated that a non-IG *MYC* partner was a survival advantage, whereas other studies found no significant difference between cases with an IG or non-IG *MYC* partner in DHL/THL [9,10,11,12,13,14]. Aukema SM et al. demonstrated that comparative copy number alteration (CNA) of DHL/THL had higher frequencies of gains at chromosome loci 8q and 12q compared with SHL (9). Next-generation target sequencing (NGS) demonstrated that the mutational profile of DHL/THL differed from that of DLBCL, NOS [16,17,18]. Gene expression profile analyses of DHL/THL revealed that DHL with *BCL2*-R (DHL-*BCL2*) and THL had germinal center B-cell-like (GCB) profiles [6,9,19,20,21]. Nevertheless, the differences between DHL/THL and SHL are still unclear.

Other than DHL/THL and SHL, we previously reported DLBCL with another abnormal *MYC* status called “DLBCL with *MYC*-cluster amplification (MCAD)” [22]. Although MCAD showed poorer clinical outcomes, the genetic features of MCAD are unclear.

In this study, we examined the genetic aspects of DHL/THL, SHL, and MCAD by targeted next-generation mutational analysis and whole-genome copy number analysis. We demonstrated that DHL/THL, SHL and MCAD had different characteristics in their genetic aspects.

## 2. Materials and Methods

### 2.1. Case Selection

We retrieved 23 patients who had DLBCL with abnormal *MYC* status (10 cases of DHL/THL, 10 cases of SHL and 3 cases of MCAD) for whole-genome copy numbers. We targeted next-generation analysis from the records of the Department of Pathology, Tokai University School of Medicine, Japan. These 23 cases were diagnosed between 2003 and 2018 and confirmed using available formalin-fixed paraffin-embedded tissues (FFPE) for molecular analysis. Some of these cases were reported in our previous study [7,15,22].

*MYC*, *BCL2*, *BCL6* rearrangements and *MYC*-cluster amplification were examined by fluorescence in situ hybridization (FISH) in all cases to identify the cases as DHL/THL, SHL, or MCAD. All clinical and laboratory data for each case and follow-up data were obtained from medical records (Appendix A Table A1 and Table A2). No patients with a clinical history of follicular lymphoma (FL) were included. This study complied with the Declaration of Helsinki for medical research involving human subjects, and institutional review board approval was previously obtained (20R-117).

### 2.2. Immunohistochemistry

Immunohistochemistry was performed using mouse monoclonal antibodies against CD3 [clone LN10, Novocastra (NV), Leica Biosystems, Tokyo, Japan], CD5 (4C7, NV), CD10 (56C6, NV), CD20 (L26, NV), BCL2 (BCL2/100/D5, NV), BCL6 (LN22, NV), MUM1 [EAU32, NV], MIB-1 (MM1, NV) and a rabbit monoclonal antibody against MYC (Y69, Abcam K.K., Tokyo, Japan) as the primary antibodies. Signal detection was performed in a Leica BOND-MAX system and the BOND Polymer Refine detection kit (DS9800, Leica Biosystems). BOND Epitope Retrieval Solution 2 (AR9640, Leica) was used for 20 min for CD3, CD5, CD10, CD20, BCL6, MUM1, MIB-1, and MYC, and 30 min for CD10 and BCL2.

A marker was positive if more than 30% of tumor cells expressed antigens for the CD3, CD5, CD10, CD20, BCL2, BCL6, and MUM1 markers. MIB-1 and MYC were semiquantitatively assessed by increments of 10%.

A thorough description of the immunohistochemical procedures is shown in Appendix B.

### 2.3. Fluorescence In Situ Hybridization (FISH)

FISH was performed using *BCL2*, *BCL6*, and *MYC* break-apart probes (Y5407, Y5408, and Y5410, respectively, Dako K.K., Vysis, Abbott Molecular, Tokyo, Japan) and a *MYC*/*IGH* fusion probe (LSI *IGH*/*MYC*/*CEP8*, Vysis, Abbot Molecular, Tokyo, Japan), and evaluated using a fluorescence microscope (Olympus BX51, Olympus K.K., Tokyo, Japan). Signals were counted in at least 100 medium- to large-sized cells, and the positivity threshold was set at 10%.

A thorough description of the FISH procedures is shown in Appendix C.

### 2.4. Whole-Genome Copy Number Analysis

The OncoScan platform (Thermo Fisher Scientific, Waltham, MA, USA) was used as we previously described [23], including the same parameters for copy number analysis identification and the minimal common regions (MCRs) of gains and losses. In summary, genomic DNA was extracted from FFPEs (QIAamp DNA Micro Kit, Qiagen K.K., Tokyo, Japan), checked for quality (EuroClonality/BIOMED-2 guidelines), and a Qubit assay was performed to measure dsDNA (Thermo Fisher Scientific K.K., Tokyo, Japan). All cases had a fragment size of at least 300 bp. Assay, visualization, and data analyses were performed under the Standard Analysis Setup using the Affymetrix GeneChip^®^ System 3000, Transcriptome Analysis Console (TAC) 4.0.2 (Appliedbiosystems, Thermo Fisher Scientific), and Multi-Sample Viewer 1.1.0.11. The NetAffx Build 20220301 (hg38) was used. Nonparametric tests were used to compare copy number alterations between groups.

A thorough description of the OncoScan procedures is shown in Appendix D.

### 2.5. Targeted Next-Generation Sequencing (NGS)

An aliquot of the same DNA extracted from FFPE and used in the OncoScan assay was also to the targeted NGS. A gene panel of 115 genes involved in aggressive B-cell lymphomas was designed (Appendix E). The procedure included a Sureselect XT Library Prep protocol using a custom SureSelect XT panel (Agilent, Santa Clara, CA, USA) and 2 × 131 bp sequencing using the MiSeq kit 600 cycles v3. (Illumina, San Diego, CA 92122, USA).

As previously described, a bioinformatic analysis was performed to evaluate the mutational landscape and tumor burden of DLBCL [24]. The summarized analysis workflow was as follows: the trimming of raw NGS reads (FASTQ), mapping, variant calling (using five callers), variant annotation, and filtering. Mutations identified by at least three different algorithms were highly confident and used in downstream analyses [24]. The criteria for somatic mutations included the following: (1) confirmed as somatic using the COSMIC database; (2) described in the literature as a somatic/driver mutation; (3) truncating (including frameshift, splicing, and stop gained); and (4) if missense, the result of damage in at least 2 of the 4 predictor software programs used (PolyPhen2, SIFT, CADD, and Mutation Assessor). In the analysis, changes were classified as mutations, likely SNPs, SNPs, and likely non-functional. Only changes classified as “mutations” were selected. The coverage of *BCL6* included exons 3–10. The mutation “load” or “burden” was calculated as the number of genes in the panel with at least one mutation. Of note, this is not a calculation based on mutations per megabase. A comparison of the means between DHL/THL and SHL was performed using conventional statistics.

### 2.6. Statistical Analysis

Statistical analyses were performed using IBM SPSS Statistics (version 27) according to the software manufacturer’s instructions (IBM Japan Ltd., Tokyo, Japan). Comparisons between groups included cross-tabulations with the chi-square test (with the likelihood ratio and Fisher’s test) and nonparametric tests (the Mann–Whitney U and/or Kruskal–Wallis H tests). The survival analysis included the Kaplan–Meier method with the log-rank test and Cox’s regression analysis.

We defined the “favorable prognosis group” as patients showing a complete response (CR) to the first course of chemotherapy and no relapse during the observation period, and the “adverse prognosis group” as patients not showing CR to the first course of chemotherapy or relapse after the first CR during the observation period.

## 3. Results

### 3.1. Clinical and Pathological Features

Clinicopathological data of the 23 cases are summarized in Appendix A. The 23 cases of DHL/THL, SHL, and MCAD histologically showed diffuse proliferation of medium to large lymphoma cells. No differences were found regarding age, sex, cell-of-origin classification (Hans), and the international prognostic index between the three subtypes. *MYC* translocation to *IGH* was observed in 40% of DHL/THL, and 70% of SHL. *MYC*-cluster amplification was detected when more than 10 signals of *MYC* were observed (Appendix F).

In the 23 cases, the variables with prognostic values were the IPI (Hazard Risk = 3.4, *p* = 0.038) and only marginally, the cell-of-origin (HR = 2.8, *p* = 0.081). Interestingly, in the SHL and DHL/THL groups, the absence of *MYC*/*IGH* fusion was associated with the clinical response to treatment (Complete response or partial response vs. other, Fisher’s Exact Test, *p* = 0.014).

### 3.2. Whole-Genome Copy Number Profiles

**Figure 1:**
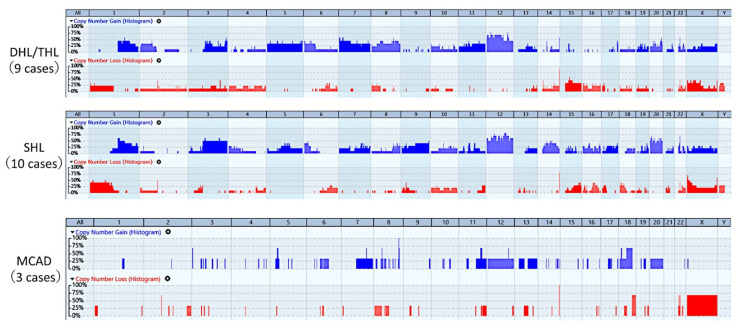
Copy number alterations of diffuse large B-cell lymphoma with abnormal MYC status. Copy number alteration profiles are similar between DHL/THL and SHL and both subtypes share the same regions of gains (blue histogram) and losses (red histogram). Gains in chromosomes 9 and 16 are more frequent in SHL. DHL/THL have larger areas with losses at chromosomes 2 to 4, although at a low frequency. Copy number alterations in MCAD are fewer than those in DHL/THL and SHL. The gain at 8q24 reflected the FISH results.

The CNA profiles were available for 22 of 23 cases (9 cases of DHL/THL, 10 cases of SHL and 3 cases of MCAD) (Figure 1).

The most relevant minimal common regions (MCRs) of genomic gains (+) and losses (−) of DHL/THL were −1p36.32 (33%), +1q25.2 (56%), +3q29 (56%), +chr.5 (44%), +6p25.3 (44%), −6q22.1 (33%), +chr.7 (44%) with a peak at 7p22.3 (56%), +chr8 (44%) with a peak at 8q23.3 (56%), +chr.12 (67%) with a peak at +12p12.2 (67%), and +20q13.33 (56%).

The most relevant MCRs of genomic gains and losses of SHL were located at −1p36.32−q21.1 (40%) with a peak at 1p31.1 (50%), +3p13−q29 (50%) with a peak at 3q26.1 (60%), +6p25.3−p22.3 (40%), −6q22−q23 (30%), +9q21.11−q34.3 (40%), +chr. 12 (50%–80%), +14q11.2−q31.1 (40%), −15q22.31 (40%), −18p11.31−p11.23 (40%), and +chr.20 (50%).

An inspection of the whole-genome view revealed that CNA profiles between DHL/THL and SHL were similar. DHL/THL and SHL both shared regions of gains and losses, except for gains in chromosomes 9p13.3−q34.3 (*p* = 0.03) and 16p11.2 (*p* = 0.01), frequently observed in SHL. DHL/THL had larger areas with losses at chr.2 to 4 at a low frequency.

There were fewer genomic alterations for MCAD than DHL/THL and SHL, but all three cases showed +8q24, which reflected the FISH result.

### 3.3. Mutational Profile with Targeted NGS

**Figure 2:**
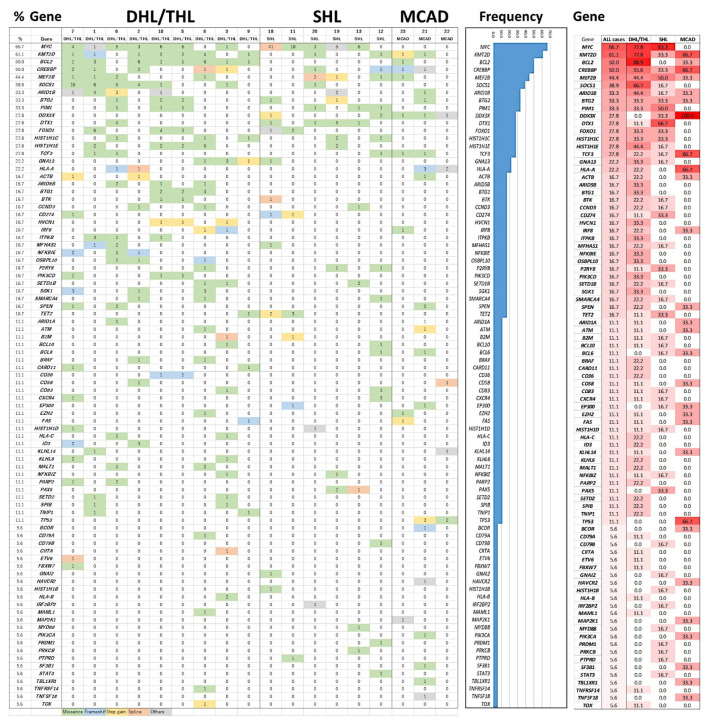
Targeted next-generation mutational profile. The most frequently mutated genes in all cases were *MYC*, *KMT2D*, *BCL2*, and *CREBBP*. The total number of mutations per gene is shown. Missence, green; frameshift, blue; stop gain, yellow; splice, red; and others, grey. On the right, the percentage of cases with mutation is shown in all cases and each group.

NGS data were available for 18 of 23 cases (9 cases of DHL/THL, 6 cases of SHL and 3 cases of MCAD) (Figure 2). Mutations were identified in 87 of the 115 genes analyzed (76%). The most frequent types of mutations were missense variants, frameshift variants, and stop gains (Figure 2).

The mutational profile of DHL/THL was characterized by a high frequency of mutations in *BCL2* (8 of 9 cases, 89%), *MYC*, and *KMT2D* (78%), *SOCS1* (67%), *CREBBP* (56%), *MEF2B*, *ARID1B*, and *HIST1H1E* (44%). The mutation “burden” was higher in the DHL/THL than in the SHL group 17 vs. 10, *p* = 0.01.

The mutational profile of SHL was characterized by a high frequency of mutations in *MYC* (83%), *DTX1* (67%), *MEF2B* and *PIM1* (50%).

DHL/THL was generally characterized by a higher mutation frequency than SHL (185.2% vs. 81.5%). The mutational profiles of DHL/THL and SHL had similarities and differences. The most relevant genes were *BCL2* for DHL/THL (89% vs. 0%, *p* = 0.001, Fisher’s Exact test) and *DTX1* for SHL (66.7% vs. 11.1%, *p* = 0.023). Statistical analyses of the two groups also highlighted the following: DHL/THL-associated genes were *KMT2D* and *SOCS1*, while SHL-associated genes were *DTX1* and *PAX5*.

The mutational profile of MCAD was characterized by *DDX3X* (100%), *KMT2D* (67%), *CREBBP* (67%), and *TP53* (67%). MCAD did not show a mutation in *MYC* (0%).

A correlation between *MYC*/*IGH* fusion, response to treatment, and mutational profile are shown in Appendix H. In summary, cases with *MYC*/*IGH* fusion were characterized by worse clinical response to treatment and a different mutational profile.

### 3.4. Combining Copy Number Alterations (CNA) and NGS Mutational Profiling

**Figure 3:**
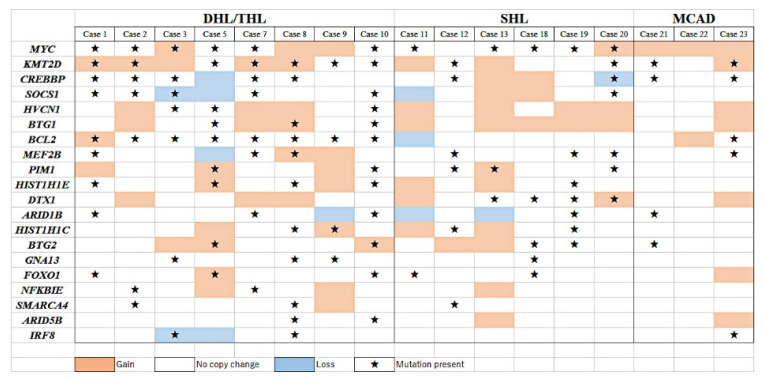
Combined copy number alterations and mutational profile.

Combining CNA and NGS data is summarized in Figure 3. A comparison between DHL/THL (eight cases), SHL (six cases), and MCAD (three cases) was made with the integration of CNA and NGS mutational profiles. DHL/THL cases had gene alterations in *MYC*, *KMT2D* and *BCL2*. However, SHL did not show changes in *BCL2.* In DHL/THL, *BCL2* showed eight mutations and one gain, whereas for SHL, there was only one loss. *KMT2D* in DHL/THL showed seven mutations and five gains, whereas SHL showed two mutations and two gains. *MYC* showed six mutations and three gains in DHL/THL, whereas for SHL, there were five mutations and one gain. All MCAD cases had a gain of *MYC* without mutation, and two of three cases had gene alteration of *KMT2D* and *BCL2* (Figure 3).

## 4. Discussion

In this study, we evaluated 23 cases of DHL/THL, SHL, and MCAD for whole-genome copy number alterations and targeted NGS to clarify differences among them.

The mutational profiles of DHL/THL and SHL had both similarities and differences. *KMT2D*, *SOCS1*, and *BCL2* were frequently mutated in DHL/THL but not in SHL. Previously reported NGS data of DHL/THL showed *CREBBP*, *BCL2*, *KMT2D*, *MYC*, *EZH2*, *IGLL5*, *FOXO1*, *SOCS1* and *SI* are frequently mutated genes [16,17,18]. Evrard et al. stated that for DHL/THL, the most frequently mutated genes were like those reported in DLBCL NOS, especially in GCB-DLBCL, but the percentage of DHL/THL with mutations on eight genes (*CREBBP*, *BCL2*, *KMT2D*, *MYC*, *EZH2*, *IGLL5*, *FOXO1* and *SOCS1*) was significantly higher than that of reported DLBCL, NOS [16]. The difference in mutation profile among DHL-BCL2, DHL-BCL6, and THL is also described; *CREBBP* and *BCL2* were frequently mutated in both DHL-BCL2 and THL cases but not mutated in DHL-BCL6 [16], and DHL-BCL6 also had a low frequency of *EZH2* mutations but did have *UBE2A* mutation [16,18]. In our study, *EZH2* was mutated in only one of seven cases for DHL-BCL2, and no mutation of *CREBBP*, *BCL2*, *EZH2* and *UBE2A* in the case of DHL-BCL. The reason is not clear, but it may be related to racial variation; therefore, analyses of more cases are required.

Epigenetic alterations relating to histone methylation (*KMT2D*, *KMT2C*, *EZH2*), histone acetylation (*CREBBP*, *EP300*), and DNA methylation (*TET2*) play an important role in tumor progression for FL and DLBCL. *KMT2D* is lysine-specific histone methyltransferase and *KMT2D* mutations may promote malignant growth by perturbing the expression of tumor suppressor genes that control B-cell activating pathways [25].

*DDX3X* was mutated in both SHL and MCAD. Cucco F. et al. reported that mutation of *DDX3X* is also observed not only in SHL but also in DHL/THL [26]. The mutation of *DDX3X* has been reported in Burkitt lymphoma, chronic lymphocytic leukemia, and natural killer T-cell lymphoma [27,28,29,30,31,32,33,34,35]. The role of *DDX3X* in malignancy remains controversial, and it has been classified as both a tumor suppressor and an oncogene [36,37]. Gong C et al. suggested that *DDX3X* promotes the translation of mRNA encoding components of the core translational machinery, thereby driving global protein synthesis [27]. In our study, the mutation of *DDX3X* is not observed in DHL/THL; therefore, *DDX3X* may not play an important role in DHL/THL. Interestingly, the NGS of a DH-FL case was reported to show mutations in *KMT2D*, *CREBBP*, *BCL2* and *MYC*, indicating a similar mutation pattern to that of DHL/THL [17].

Therefore, the mutation profiles of DHL/THL and SHL seem to be different because of their different oncogenesis; DHL/THL is suggested to have a mutation profile occurring after *BCL2* rearrangements.

We demonstrated that the copy number patterns of DHL/THL and SHL were similar, supporting a previous study [9]. Both were characterized by −1p, +3q, +5q, +6p, −6q, +chr.12, −15q, and +chr.20 changes, but gains at 9p13.3−q34.3 and 16p11.2 were more frequently observed in SHL (*p* = 0.03 and 0.01, respectively).

When we divided the DHL/THL and SHL cases into “favorable prognosis group” and the “adverse prognosis group” according to our previous study [15], there were some differences between the two groups by CNA and NGS (Appendix G). Copy number gain at 3q11.2 occurred in the favorable prognosis group (*p* = 0.012). Mutation in *TCF3* was associated with the adverse prognosis group (*p* = 0.035) (Appendix A Table A1 and Table A2 and Appendix G).

Clinicopathological and genetic features of MCAD is unclear due to a few reports [22,38]. We evaluated three cases of MCAD for whole-genome CNA and targeted NGS to compare with DHL/THL and SHL. In this study, the CNA of MCAD showed fewer alterations than for DHL/THL and SHL. The NGS of all the MCAD cases had no MYC mutation, but only in MCAD was *TP53* mutated, suggesting that MCAD is genetically different from DHL/THL and SHL. Larger series of cases will be necessary to validate these results.

Other groups previously described the molecular classification of DLBCL [39,40,41,42]. According to these molecular classifications [39,40,41], the DHL/THL in our study, which had *BCL2*, *KMT2D*, and *CREBBP* mutations, belongs to the *EZB/C3* groups. SHL belongs to the other (nonsubtyped) group, as it had low-frequency mutations (1/6 of cases or 2/6 of cases) of *MYD88*, *CD274*, *PRKCB*, *STAT3*, *P2RY8*, *KMT2D*, *CREBBP*, and *EP300* [39,42]. MCAD belongs to the composite group, as it had high-frequency mutations (2/3 of cases) of *TP53*, *KMT2D*, and *CREBBP* [39,42]. Of note, our study used a custom gene panel, so the inclusion in these groups is not direct.

This project analyzed 23 cases of DLBCL, including 10 cases corresponded to single-hit lymphoma (SHL), 10 cases to double and triple-hit lymphoma (DHL/THL), and 3 cases to MYC-cluster amplification (MCAD). The number of cases is a limitation. Therefore, a larger series of cases will be analyzed in the future.

Tumor mutational burden (TMB) is defined as the total number of nonsynonymous mutations per coding area of a tumor genome. Initially, it was determined using whole-exome sequencing, but due to the high cost and long turnaround time of this method, targeted panel sequencing is currently being explored to measure TMB [43]. Based on 115 genes of aggressive B-cell lymphomas, the NGS profiling characterized DHL/THL with a higher mutation burden than SHL.

There have been advances in the understanding of the pathogenesis of DLBCL. The work of Chapuy B et al. analyzed a series of 304 DLBCL cases from different institutions using whole-exome sequencing (WES) [41]. The copy number analysis was estimated on the panel using ReCapSeg and GISTIC. The *BCL2*, *BCL6*, and *MYC* rearrangements were not assessed by FISH but by WES. Schmitz R et al. [40] analyzed 574 DLBCL cases using exome and transcriptome sequencing, array-based DNA copy number analysis, and targeted amplicon resequencing of 372 genes to identify genes with recurrent aberrations. This research focused more on the distinction between ABC and GCB subtypes. Nevertheless, it doesn’t provide information about the rearrangement of *MYC*, *BCL2*, and *BCL6*. Similar to the work of Chapuy, Wright GW made a probabilistic classification of DLBCL, and several subtypes were identified: *MCD*, *BN2*, *N1*, *EZB*, *ST2*, and *A53* [42].

Recently, an update to the lymphoma classification has been released. In the International Consensus Classification of Mature Lymphoid Neoplasms by Campo E et al. [44], The high-grade B-cell lymphomas (HGBCL-DH) now comprise two groups: HGBCL with *MYC* and *BCL2* rearrangements (with or without *BCL6* rearrangement) (HGBCL-DH-BCL2) and a new provisional entity, HBGBL with *MYC* and *BCL6* rearrangements (HGBCL-DH-BCL6). In the fifth edition of the World Health Organization Classification of Haematolymphoid Tumours: Lymphoid Neoplasms [45], an algorithm to diagnose the different entities is shown, including DLBCL NOS, Burkitt lymphoma, HGBL NOS, HGBL-11q, and DLBCL/HGBL-MYC/BCL2. In this new classification, the lymphoma subtype of this research is named diffuse large B-cell lymphoma/high-grade B-cell lymphoma with *MYC* and *BCL2* rearrangements, and *BCL6* is no longer included. The molecular characteristics are not described in both the ICC and the WHO classifications. Therefore, the field of study of the clinicopathological characteristics and genomic profile of high-grade B-cell lymphoma remains in development.

*MYC*-cluster amplification in DLBCL is a rare finding. We previously described the clinicopathological characteristics and whole-genome copy number of one case [22]. In this research, we expand to three new cases, including the characterization of the mutational landscape.

In this research, we defined favorable and adverse prognosis groups. We defined the “favorable prognosis group” as patients showing a complete response (CR) to the first course of chemotherapy and no relapse during the observation period, and the “adverse prognosis group” as patients not showing CR to the first course of chemotherapy or relapse after the first CR during the observation period. We used this prognostic classification in a recent publication [46,47,48,49]. This publication is relevant because we characterized the gene expression profile of HGBCL using the Lymph2Cx assay and a pan-cancer immune profiling panel (LBL-10043-08). Several genes were overexpressed in the DHL/THL adverse group, including *AICDA*, *LILRB1*, *CD70*, *NUP107*, *CXCL11*, *ADORA2A*, and *RELA*. We concluded that *AICDA* and AID could be predictors of an adverse clinical outcome in DHL/THL and immunohistochemistry of AID was useful in finding DHL/THL-adverse prognosis group [15]. Of note, AICDA plays a relevant pathogenic role in lymphoma, as we have recently highlighted [46,47,48,49].

The mutational profiling highlighted a series of genes, and their functions and roles are summarized in Table 1. In summary, these genes were proto-oncogenes or tumor suppressor genes with roles in the cell cycle, apoptosis, DNA repair, transcription, metabolic changes, and antigen presentation. Therefore, the presence of damaging mutations in these genes are expected to affect these cancer pathways. Mutational profiling of aggressive B-cell lymphomas has identified numerous genes that are involved in but not exclusive to, certain diagnostic categories [50]. In DLBCL, at least 10% of the cases identify around 15 mutations, with a long tail of infrequent mutations [50]. A recent analysis of large series of DLBCL has shown that the most frequently mutated genes in DLBCL included *KMT2D*, *MYD88*, *CREBBP*, and *TP53* [51], and *BCL2*, *HIST1H1E*, and *PIM1* [52]. Our series of aggressive lymphomas also had mutations in these genes. Of note, there are currently nine genes considered actionable (*CDK6*, *TP53*, *CDKN2A*, *PTEN*, *MYC*, *ARID1A* and *CD79B*, *EZH2*, and *NOTCH1*) as potential therapeutic targets of drugs in early clinical trials [51].

Recently, other researchers have analyzed the clinicopathological characteristics of high-grade B-cell lymphomas.

Chulin Sha et al. screened 928 patients and identified a group of 83 patients that was named “molecular high-grade B-cell lymphoma (MHG)” [19]. In approximately half of these 83 cases (i.e., approximately 40 cases), rearranged *MYC* and double hits were identified [19]. That group of 83 patients was characterized by a GCB phenotype and a gene expression signature of proliferation. MHG had a high frequency of mutations of *KMT2D* and *BCL2* (>40%) and a lower extent also mutations of *TP53*, *TNFRSF14*, *EZH2*, *MYC*, *CREBBP*, and *SOCS1* [19]. Our mutational data is comparable. Nevertheless, since our research, we followed the current 2016 WHO lymphoma classification, a direct comparison with the MHG group cannot be made.

Scott D.W. et al. analyzed 1228 DLBCL biopsies, and in 7.9% of the cases, the high-grade B-cell lymphoma with *MYC* and *BCL2* and/or *BCL6* rearrangements (HGBL-DH/TH) was made. The frequency of HGBL-DH/TH was more frequent in the GCB phenotype, as in our study. Nevertheless, Scott DW and colleagues did not analyze the whole-genome copy number and mutational profile [20].

Künstner A. et al. recently described the mutational landscape of high-grade B-cell lymphoma with *MYC*-, *BCL2* and/or *BCL6* rearrangements using whole-exome sequencing [18]. This research analyzed 47 clinically annotated cases of HGBL, including 21 DHL-BCL2, 17 DHL-BCL6, and 9 THL. In general, the most frequently mutated genes were *CREBBP* and *KMT2D* [18]. This is comparable to our research. Nevertheless, our project had different groups: DHL/THL, SHL, and MYC-cluster amplification (MCAD). As a result, our data provide a different approach to the mutational landscape of HGBL.

Tsai C.C. has recently analyzed 282 cases of DLBCL and identified 47 (16.7%) with *MYC* translocation, which included 24 DH/THL [53]. A total of 62.5% of the DH/THL were GCB, and DH/THL cases were associated with an unfavorable overall survival [53]. This research is similar to our project. Nevertheless, it lacks a whole-genome copy number and mutational profiling.

Zeng D et al. published a review manuscript regarding high-grade B-cell lymphoma with rearrangement of *MYC* and *BCL2* and/or *BCL6*, also known as double-hit lymphoma (DHL) [54]. This review concluded that most of the relapsed or refractory LBCL belonged to this WHO subtype. Our research focused only on this subtype and approximately 50% of the cases did not achieve a clinical response. We did not compare with DLBCL, which was not otherwise specified. Nevertheless, we have recently shown that cases with high gene expression of *BCL2*, *MYC*, and *ENO3* are associated with an unfavorable overall survival of the patients [47,55,56], and that HGBL accounts for around 10% of the cases [46,47,48,49,50,51,52,53] and is characterized by higher CD163 but lower PTX3 expression [57]. Therefore, this WHO subtype is being characterized progressively.

This research characterized the mutational landscape of HGBL, and we found that the most frequently mutated genes were *MYC*, *KMT2D*, *BCL2*, *CREBBP*, *MEF2B*, *SOCS1*, *ARID1B*, *BTG2*, and *PIM1*. Bolen C.R. et al. recently described a series of somatic mutations with prognostic impact in DLBCL NOS [58]. Among them, the most relevant was *BCL2* (Hazard Risk = 2.2, *p* = 0.0025). Other genes of our series were also mutated in the Bolen C.R. series but had not prognostic relevance. Therefore, HGBL and DLBCL NOS share a common mutational profile, but all the data together indicate that HGBL has different characteristics.

## 5. Conclusions

Integrating copy number change and mutational profile of DHL/THL, SHL, and MCAD showed different characteristics in each other.

## Figures and Tables

**Table 1:** Function and role of the most relevant mutated genes.

Gene	Function
*MYC*	Proto-oncogene and transcription factor that activates the transcription of growth-related genes
*KMT2D*	Histone methyltransferase with role in chromatin remodeling and DNA repair
*BCL2*	Apoptosis regulator (suppressor)
*CREBBP (CBP)*	Acyltransferase with a role in the acetylation of histones and non-histone proteins, chromatin remodeling, and transcriptional co-activation of different transcription factors
*MEF2B*	DNA binding protein, gene expression regulator.
*SOCS1*	Negative regulator of type I and II interferon signaling and other cytokines
*ARID1B*	Transcriptional activation and repression of select genes by chromatin remodeling, and cell cycle activation
*BTG2*	Antiproliferative protein, regulation of B1/S transition of the cell cycle
*PIM1*	Proto-oncogene with serine/threonine kinase activity, and involved in cell survival and cell proliferation
*DDX3X*	Multifunctional ATP-dependent RNA helicase. Role in transcriptional regulation
*DTX1*	Ubiquitin ligase acts as a positive regulator of the Notch-signaling pathway (cell-cell communication, and cell-fate determination)
*TCF3*	Critical role in lymphopoiesis, both B and T lymphocyte development
*HLA-A*	Antigen-presenting major histocompatibility complex class I (MHCI) molecule
*TP53*	Tumor suppressor in many tumor cells, cell cycle arrest, apoptosis, senescence, DNA repair, and metabolism changes.

Based on UniProt and GeneCards.

**Figure A1:**
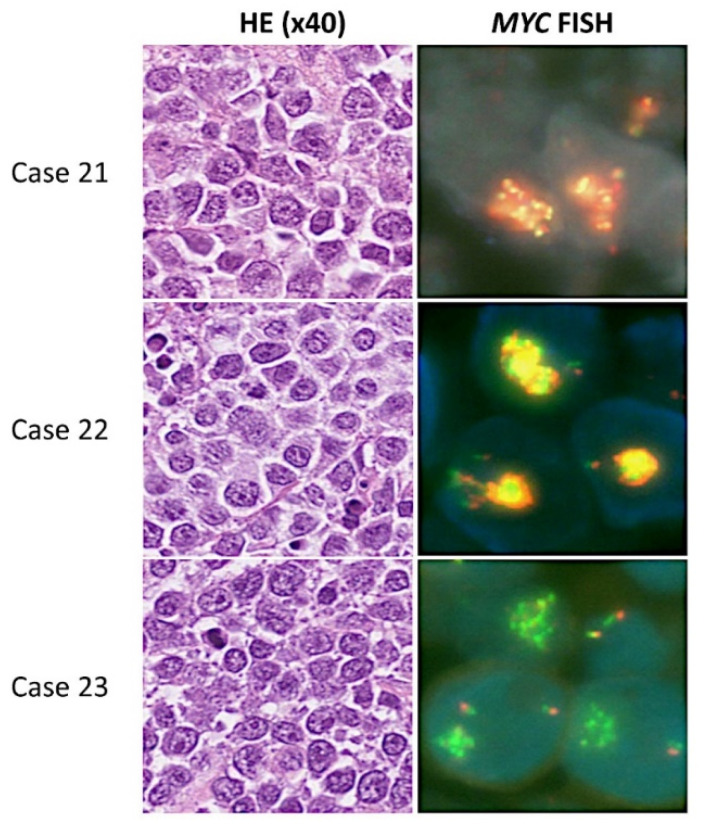
Histology and FISH of diffuse large B-cell lymphoma with MYC-cluster amplifica-tion (MCAD).

**Figure A2:**
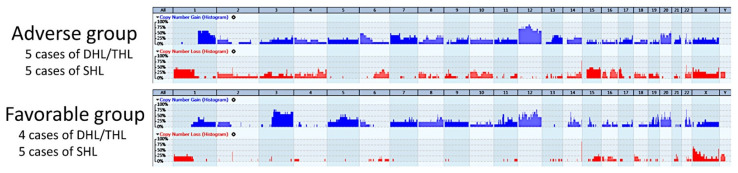
Copy number alterations of the adverse group and favorable group.

**Figure A3:**
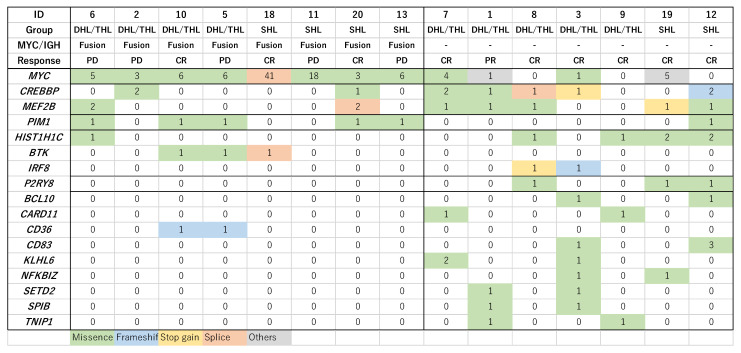
Correlation between *MYC*/*IGH*, response to treatment, and mutational profile. Missence, green; frameshift, blue; stop gain, yellow; splice, red; and others, grey. PD—progressive disease; CR—complete response; PR—partial response.

## Data Availability

All data is available upon request to Joaquim Carreras (joaquim.carreras@tokai-u.jp).

## Appendix A

**Table A1:** Clinicopathological data of the 23 cases.

Id.	Subtype	Age	Gender	LDH	Stage	PS	BM	IPI	Therapy	Response to 1st Chemotherapy	OS	D/A	Prognosis
1	DHL/THL	69	F	+	4	0	−	High	R-CHOP	Partial response	69	Dead	Adverse
2	DHL/THL	63	F	+	4	2	+	High	R-Hyper-CVAD	Progressive disease	4	Dead	Adverse
3	DHL/THL	67	M	+	4	4	−	High	R-COP	Relapse after 1st CR	13	Dead	Adverse
4	DHL/THL	78	M	−	2	0	−	Low	R-CHOP	Relapse after 1st CR	55	Dead	Adverse
5	DHL/THL	55	F	+	4	0	−	Low-intermediate	R-Hyper-CVAD	Progressive disease	27	Dead	Adverse
6	DHL/THL	73	F	+	2	0	−	Low-intermediate	DA-EPOCH-R	Progressive disease	8	Dead	Adverse
7	DHL/THL	49	F	No data	2	0	−	Low	R-CHOP	Complete response	127	Alive	Favorable
8	DHL/THL	66	M	+	2	0	−	Low-intermediate	R-CHOP	Complete response	126	Alive	Favorable
9	DHL/THL	61	F	+	4	0	−	High	R-CHP	Complete response	97	Alive	Favorable
10	DHL/THL	57	M	No data	4	0	+	Low	R-CHOP→DA-R-EPOCH	Complete response	33	Alive	Favorable
11	SHL	67	F	+	2	0	−	Low-intermediate	R-CHOP	Progressive disease	10	Dead	Adverse
12	SHL	83	F	+	3	1	−	High-intermediate	R-COP	Relapse after 1st CR	23	Dead	Adverse
13	SHL	68	M	+	4	0	+	High-intermediate	R-CHOP	Progressive disease	32	Dead	Adverse
14	SHL	53	M	+	2	0	−	Low-intermediate	R-CHO	Relapse after 1st PR	12	Dead	Adverse
15	SHL	66	F	+	4	0	+	High	DA-EPOCH-R	Progressive disease	6	Dead	Adverse
16	SHL	62	M	+	2	0	−	Low-intermediate	R-CHOP	Complete response	167	Alive	Favorable
17	SHL	62	M	−	1	0	−	Low	R-CHOP	Complete response	109	Alive	Favorable
18	SHL	76	M	+	2	1	No data	Low	R-CHOP	Complete response	88	Alive	Favorable
19	SHL	58	F	−	3	0	−	Low-intermediate	R-CHOP	Complete response	105	Alive	Favorable
20	SHL	41	F	−	2	0	−	Low	R-CHOP	Complete response	99	Alive	Favorable
21	MCAD	51	M	+	3	1	−	Low-intermediate	EPOCH-R	Partial response	50	Alive	N/A
22	MCAD	72	M	+	4	1	−	High	R-CHOP	Progressive disease	6	Dead	N/A
23	MCAD	70	M	+	4	2	−	High	MTX+R-CHOP	Partial response	14	Alive	N/A

PS—performance status; BM—bone marrow infiltration; IPI—international prognostic index; OS—overall survival (months); D/A—overall survival outcome (dead vs. alive).

**Table A2:** Clinicopathological data of the 23 cases (continuation).

Id.	Subtype	MYC F	MYC/IGH F	BCL2 F	BCL6 F	COO	MYC (%)	MIB-1 (%)	CD3	CD5	CD10	CD20	BCL2	BCL6	MUM1	CNA	NGS
1	DHL/THL	+	−	+	−	non-GCB	70	50	−	−	−	+	+	−	−	+	+
2	DHL/THL	+	+	+	+	GCB	90	60	−	−	+	+	+	+	−	+	+
3	DHL/THL	+	−	+	No signal	non-GCB	90	70	−	−	−	+	+	+	+	+	+
4	DHL/THL	+	−	−	+	non-GCB	30	80	−	−	−	+	−	−	+	+	No data
5	DHL/THL	+	+	+	+	GCB	90	80	−	−	+	+	+	+	−	+	+
6	DHL/THL	+	+	−	+	non-GCB	10	90	−	−	−	+	+	+	+	No data	+
7	DHL/THL	+	−	+	−	GCB	70	80	−	−	+	+	−	+	−	+	+
8	DHL/THL	+	−	+	−	GCB	80	80	−	−	+	+	−	+	−	+	+
9	DHL/THL	+	−	+	+	GCB	80	70	−	−	+	+	+	+	−	+	+
10	DHL/THL	+	+	+	+	GCB	80	80	−	−	+	+	+	+	−	+	+
11	SHL	+	+	−	−	GCB	100	90	−	−	+	+	−	+	−	+	+
12	SHL	+	−	−	−	non-GCB	80	90	−	−	−	+	+	+	+	+	+
13	SHL	+	+	−	−	non-GCB	60	70	−	−	−	+	+	−	+	+	+
14	SHL	+	+	−	−	non-GCB	80	90	−	−	−	+	+	−	+	+	No data
15	SHL	+	+	−	−	GCB	80	90	−	+	+	+	+	−	+	+	No data
16	SHL	+	+	−	−	GCB	90	80	−	−	+	+	−	−	−	+	No data
17	SHL	+	−	−	−	non-GCB	50	100	−	−	−	+	+	+	+	+	No data
18	SHL	+	+	−	−	GCB	90	90	−	−	−	+	−	+	−	+	+
19	SHL	+	−	−	−	GCB	60	90	−	−	+	+	−	+	−	+	+
20	SHL	+	+	−	−	GCB	50	80	−	−	+	+	−	+	+	+	+
21	MCAD	−	No data	−	−	GCB	90	90	−	−	+	+	−	+	+	+	+
22	MCAD	−	No data	−	−	GCB	80	90	−	−	+	+	−	+	−	+	+
23	MCAD	−	No data	+	−	GCB	80	80	−	−	+	+	+	+	−	+	+

F—FISH; COO—cell-of-origin subtypes (Hans’ classifier); CNA—whole-genome copy number analysis (OncoScan); NGS—next-generation mutational analysis.

## Appendix B. Immunohistochemical Procedures

Immunohistochemical staining procedures were carried out in a BOND-MAX fully automated immunohistochemistry, and in situ hybridization staining system (Leica Biosystems, Tokyo, Japan), using the Bond Polymer Refine Detection (DS9800). The primary antibodies were purchased from Novocastra/Leica and were the following: BCL2 (clone bcl-2/100/D5), BCL6 (LN22), CD10 (56C6), CD20 (L26), CD3 (LN10), CD5 (4C7; NV), MIB-1 (KI67-MM1-L-CE), MUM1 (EAU32), MYC (Y69, Abcam K.K., Tokyo, Japan). Epstein–Barr virus-encoded small RNAs (EBER) were detected with Bond ready-to-use ISH EBER probe (PB0589). All antibodies but Ki67 used the BOND Epitope Retrieval Solution 2 (AR9640); Ki67 used the BOND Epitope Retrieval Solution 1 (AR9961).

## Appendix C. Fluorescence In Situ Hybridization (FISH) Procedures

FISH was performed using commercial DNA FISH probes targeting *BCL2*, *BCL6*, and *MYC* (break-apart probes Y5407, Y5408, and Y5410, respectively, Dako K.K., Tokyo, Japan), and a *MYC*/*IGH* fusion probe (LSI IGH/MYC/CEP8, Vysis, Abbot Molecular, Tokyo, Japan). Slides were deparaffinized in xylene and hydrated in decreasing ethanol solutions. Pretreatment consisted of heating in Milli-Q water in a pressure cooker (RC-12 Cooker; Asahi Light Metal Industry Co., Ltd., Osaka, Japan) inside a microwave (NE-EH229; Panasonic K.K., Osaka, Japan) for 3 min at maximum pressure; transferred to a 37 °C pre-warmed pepsin solution in 0.01 m HCl and incubated for 10 min (pepsin, P7012-250 MG; Sigma-Aldrich; 1 mol/l hydrochloric acid, 083-01095; Wako, Neuss, Germany). Once denatured at 85 °C for 5 min, overnight hybridization was performed at 45 °C (DakoCytomation hybridizer). The washing steps consisted of 0.4 × SSC/0.3% IGEPAL at 72 °C for 2 min, 2 × SSC/0.1% IGEPAL at room temperature for 1 min and 2 × SSC at room temperature for 5 min (UltraPure™ 20 × SSC buffer, 15557-044; Invitrogen; IGEPAL^®^CA-630, I3021-50ML; Sigma-Aldrich). Nuclear counterstain was performed with DAPI, and slides were mounted with immunofluorescent media (DAPI-II Counterstain; Vysis). The FISH signals were assessed first in control lymphoid tissue and later in the lymphoma cases under a fluorescent microscope BX53, DP73 camera, and cellSens software (Olympus K.K., Tokyo, Japan).

## Appendix D. Whole-Genome Copy Number Analysis Using OncoScan Platform

Slides containing at least 70% of lymphoma cells based on histology and immunohistochemistry were selected for DNA extraction from formalin-fixed paraffin-embedded (FFPE) tissue sections (QIAamp DNA Micro Kit, 56304; Qiagen K.K, Tokyo, Japan), and resuspended in ATE buffer. DNA concentration was measured by Qubit Fluorometric Quantification (Thermo Fisher Scientific K.K., Tokyo, Japan). All cases were assessed for quality by polymerase chain reaction (PCR) amplification following BIOMED-2 guidelines. The OncoScan microarray platform was used for integrated analysis of whole-genome copy number changes and loss-of-heterozygosity (LOH) (OncoScan^®^FFPE Assay Kit; Affymetrix/ThermoFisher, Tokyo, Japan). The array utilizes the molecular inversion probe (MIP) technology; the copy number resolution is 50–100 kb in ~900 cancer genes, 300 kb outside of the cancer genes, and has a high dynamic range of 10 + copies. The assay was performed according to the manufacturer’s instructions and under the standard analysis setup (this is not a matched normal analysis). The assay’s kit contains a negative control (TE buffer, #902249) and positive control (pool of normal DNA, #902249). The Affymetrix GeneChip System 3000 was used to the generation of the CEL files. The chromosome analysis suite (AppliedBiosystems, Thermo Fisher Scientific, version 4.3.0.71) was used for the generation of OSCHP files. Array type OncoScan and OncoScan CNV, genome version hg38 (NetAffx Build 20220301), and FFPE NA36 analysis workflow. The copy number reference model was the FFPE.ma36.r3.REF_MODEL, and the OncoScan.na36.r2.annot.db ad the annotation file. Quality control (QC) metrics included MAPD, ndSNPQC, celpaircheck status equals pass, and ndwavinessSD, among others. Each case was visualized in the chromosome analysis suite and the whole-genome view in the RHAS multisample viewer 1.1.0.11 (Applied Biosystems).

## Appendix E. List of Genes in the custom NGS Panel

*ACTB*	*ARID1A*	*ARID1B*	*ARID5B*	*ATM*	*B2M*	*BCL10*	*BCL2*	*BCL6*	*BCOR*
*BRAF*	*BTG1*	*BTG2*	*BTK*	*CARD11*	*CCND3*	*CD274*	*CD36*	*CD58*	*CD70*
*CD79A*	*CD79B*	*CD83*	*CDKN2A*	*CDKN2B*	*CIITA*	*CREBBP*	*CXCR4*	*DDX3X*	*DIS3*
*DTX1*	*EBF1*	*EP300*	*ETS1*	*ETV6*	*EZH2*	*FAS*	*FBXW7*	*FOXO1*	*GNA13*
*GNAI2*	*HIST1H1B*	*HIST1H1C*	*HIST1H1D*	*HIST1H1E*	*HLA-A*	*HLA-B*	*HLA-C*	*HVCN1*	*ID3*
*IRF2BP2*	*IRF4*	*IRF8*	*ITPKB*	*KLHL14*	*KLHL6*	*KMT2D*	*KRAS*	*MALT1*	*MAML1*
*MAP2K1*	*MAPK1*	*MCL1*	*MEF2B*	*MFHAS1*	*MIR17*92*	*MYC*	*MYD88*	*NFKBIA*	*NFKBIE*
*NFKBIZ*	*NOTCH1*	*NOTCH2*	*NRAS*	*OSBPL10*	*P2RY8*	*PARP2*	*PAX5*	*PCBP1*	*PIK3CA*
*PIK3CD*	*PIM1*	*POU2F2*	*PRDM1*	*PRKCB*	*PTEN*	*PTPRD*	*REL*	*RHOA*	*RRAGC*
*S1PR1*	*S1PR2*	*SETD1B*	*SETD2*	*SF3B1*	*SGK1*	*SMARCA4*	*SOCS1*	*SPEN*	*SPIB*
*STAT3*	*STAT6*	*TBL1XR1*	*TCF3*	*TET2*	*TMEM30A*	*TMSB4X*	*TNFAIP3*	*TNFRSF14*	*TNIP1*
*TOX*	*TP53*	*XBP1*	*XPO1*	*ZEB2*					

## Appendix F

Histology and FISH of diffuse large B-cell lymphoma with MYC-cluster amplification (MCAD). All three cases of MCAD histologically show the diffuse proliferation of medium to large lymphoma cells. In the FISH analysis, MYC-cluster amplification (more than 10 signals) was observed in each case.

## Appendix G

Copy number alterations of the adverse group and favorable group, which are composed of DHL/THL and SHL. Copy number gains of 3q11.2 were found in the favorable prognosis group (*p* = 0.012).
